# Limits to blue economy: challenges to accessing fishing livelihoods in Ghana’s port communities

**DOI:** 10.1007/s40152-023-00302-8

**Published:** 2023-03-22

**Authors:** Raymond K. Ayilu

**Affiliations:** Climate, Society & Environment Research Centre, Faculty of Arts and Social Sciences, University of Technology Sydney, Broadway, Sydney, NSW 2007 Australia

**Keywords:** Blue growth, Small-scale fishing, Coastal development, Political ecology, Displacement, Securitisation

## Abstract

The blue economy concept has drawn global attention to the maritime economy, recognising expanding maritime industries such as shipping as crucial drivers of economic growth. In recent decades, seaports have correspondingly witnessed significant expansion, allowing them to play a substantial role in achieving blue growth. This study examines the challenges faced by small-scale fishing actors in gaining access to fishing livelihoods in coastal fishing communities close to Ghanaian ports. Drawing on political ecology, the study demonstrates how securitisation in port areas and dispossession has resulted in unstable fishing livelihoods in port communities. The study shows that the growth-oriented goals of port expansions and port security measures have restricted fishing communities’ access to coastal fishing spaces and caused congestion in the canoe bays of Ghana’s fishing harbours. In addition, the urbanisation around the ports has impacted fishers’ ability to meet the rising cost of living in fishing communities with fishing incomes. Furthermore, the study discusses how the new Jamestown fishing harbour complex project has displaced small-scale fishing actors and become a site of contestation between a coastal fishing community and local government authorities. In conclusion, as coastal fishing actors lose their only source of livelihood, resistance may escalate into different forms of maritime conflicts in the blue economy. The study recommends addressing the marginalisation and exclusion of traditional coastal fishing livelihoods to ensure a more equitable blue economy.

## Introduction

The maritime economy is rapidly expanding, with much attention recently paid to traditional and emerging maritime industries through blue economy initiatives (Jentoft et al., 2022). The notion of blue growth recognises maritime economic activities as crucial drivers for maximising economic growth and employment and constitutes a strategy to ensure the long-term environmental sustainability of marine sectors (Abhinav et al., 2020; Burgess et al., 2018; Eikeset et al., 2018). The European Union (EU), for instance, proposed blue growth as a strategy to steer the EU out of the global financial crisis of 2008 by opening Europe’s oceans, seas, and coastal areas for job creation and economic growth (European Commission, 2012). In recent decades, seaport developments have been associated with the blue economy (Seisdedos & Carrasco, 2020; Tsakiridis et al., 2021), with ports witnessing significant growth, enabling them to play a more prominent role in achieving blue growth (Stanković et al., 2021).

Globally, studies have shown that the blue economy developments, such as ports, maritime zone, aquaculture, industrial parks, and eco-tourism, could potentially displace traditional fishing livelihoods and small-scale local operators (Ayilu et al., 2022; Cohen et al., 2019; Fabinyi et al., 2022; Okafor-Yarwood et al., 2020). In Ghana, small-scale fisheries research has focused on important traditional challenges around how the decline in fish stocks is linked to overexploitation, illegal fishing activities, and the activities of industrial trawlers as well as how climate change directly impacts small-scale fishing activities (Afoakwah et al., 2018; Ankrah, 2018; Atta-Mills et al., 2004; Freduah et al., 2017). This literature has made significant contributions to the field of small-scale fisheries by analysing coastal livelihoods, food security, and poverty in relation to the use and governance of marine resources and the wellbeing of fishing actors. However, the literature has provided few explanations for the blue economy’s multifaceted coastal shifts affecting urban small-scale fishing in port communities discussed in this study. In these rapidly transforming fishing communities, small-scale fishing livelihoods are entangled in complex political-economic factors relating to access and control over coastal and ocean spaces, not just declining fisheries, for which Kadfak and Oskarsson (2020) have called for theoretical consideration. While some studies in Ghana have examined the impact of emerging blue economy growth-oriented expansion on coastal fishing communities, such as oil exploration (Ackah-Baidoo, 2013; Adjei & Overå, 2019; Adusah-Karikari, 2015; Owusu, 2019; Siakwah, 2018), port development, expansions, and operations remain to be addressed (Kalina et al., 2019; Okafor-Yarwood et al., 2020).

Recent port developments and expansions in Ghana aim to modernise and unlock the country’s economic growth opportunities through maritime trade (Ghana Ports and Harbours Authority [GPHA], 2022). In line with these visions, the government has adopted neoliberal port strategies to achieve this ambitious economic development plan, including increased private sector participation and adopting international regulations and standards to improve port operational efficiency and increase trade competitiveness (GPHA, 2022). For instance, private sector investments are spearheading a USD 1.5 billion expansion of the Port of Tema and a USD 475 million expansion of the Port of Takoradi (GPHA, 2022).

Large-scale ports are multi-dimensional coastal landscapes that have lasting economic and developmental impacts on regional economies, playing a central role in providing direct and indirect employment (Alamoush et al., 2021; Olukoju, 2020). In the recent COVID-19 pandemic, ports were prominent in the global supply chain, facilitating the delivery of medicine, raw materials, food products, and energy. However, ports have also had significant negative social and environmental implications. For instance, land (re)claiming for port developments and expansion, dredging and disposal, vessel traffic and land transport activities, cargo handling, and industrial and semi-industrial operations leave significant social and environmental footprints (Alamoush et al., 2021; Bailey & Solomon, 2004). Moreover, there are social and political contestations over land appropriation, coastline privatisation, and exclusion and displacement of poor coastal populations (Fabinyi et al., 2022; Kadfak & Oskarsson, 2020; Kalina et al., 2019).

By employing political ecology notions of accumulation by securitisation and dispossession (Harvey, 2003; Massé & Lunstrum, 2016), this paper examines the impact of port development, expansions, and operations on adjacent fishing communities in Ghana. The study draws on a case study of fishing communities nearby Ghana’s main ports to understand the challenges facing small-scale fishing livelihoods in the context of port developments. It contributes to the emerging blue justice literature contesting the impacts of the blue economy (Jentoft et al., 2022) by arguing that recent maritime port developments and transformations in Ghana reflect growth-oriented expansions that exclude Indigenous coastal fishing livelihoods. The study employed a qualitative research approach using interviews with local fishing actors in port communities and with a port manager to understand how these urbanised coastal community transitions affect fishing livelihoods.

## Accumulation by securitisation and dispossession

The blue economy upsurge has turned global attention from terrestrial-based to ocean-based resources for economic and industrial purposes, with such efforts influenced by market and growth-oriented tendencies that undermine coastal people’s livelihoods (Barbesgaard, 2018; Bennett, 2019). The blue economy extends from the idea of the green economy (Barbesgaard, 2018; Silver et al., 2015) and has generated concerns over equitable distribution and justice in the utilisation of coastal and ocean environments (Bennett et al., 2019; Jentoft et al., 2022). It has therefore been a focus of political ecology research. Political ecology emerged to understand how environmental and political forces interact to mediate social and environmental change (Bassett & Peimer, 2015; Bryant, 1992; Nygren & Rikoon, 2008). Academic literature has emphasised the significance of political ecology for understanding marine resource access, exclusion and displacement, and the socio-economic struggles of poor and disadvantaged communities (Childs & Hicks, 2019;Kadfak & Oskarsson, 2020; Maharaj, 2017; Nolan et al., 2020; Quist & Nygren, 2015).

Recent political ecology scholars have critiqued dominant blue economy paradigms, linking increasing incidences of ‘ocean grabbing’ or ‘blue grabbing’ to the growth imperatives of the blue economy (Barbesgaard 2018; Bavinck et al., 2017; Benjaminsen & Bryceson, 2012; Childs & Hicks, 2019; Morrissey, 2017; Winder & Le Heron, 2017). Others have argued for blue degrowth as an alternative to the blue growth ‘growth-driven’ imperative and advocated for a more critical understanding of the concept (Ertör & Hadjimichael, 2020). In this study, two concepts within political ecology are used — accumulation by dispossession and securitisation. The study draws on these two political ecology notions to examine the impact of port developments, expansions, and operations on local fishing livelihoods in Ghana’s blue economy. In the context of rapid transitions and transformations in the blue economy (Barbesgaard, 2018; Brent et al., 2018), these conceptual framings are crucial for demonstrating the territorial enclosures occurring in Ghana’s maritime space (Bavinck et al., 2017; Bush & Marschke, 2016).

The concept of accumulation by securitisation (Massé & Lunstrum, 2016) captures how capital accumulation, often tied to land and resource enclosure, is enabled by the practices and the logic of security. State or private actors often impose securitisation logic by, for instance, declaring a specific territory a security zone, militarising it, and erecting a buffer zone to exclude others (Massé & Lunstrum, 2016). This notion of securitisation is built on the literature on ‘green grabbing’, a contemporary form of accumulation of land and natural resources and exclusion of vulnerable communities (Corson et al., 2013; Fairhead et al., 2012; Green et al., 2015). Increasingly, a growing number of political ecologists have begun to examine the securitisation of the blue economy as a form of market-oriented expansion and accumulation in the maritime space (Barbesgaard, 2018; Barbesgaard, 2019; Brent et al., 2018; Kalina et al., 2019; Zhang & Bateman, 2017). This growing body of research demonstrates that the processes governing access to the ocean and coastal spaces are structured in complex power relations within a politicised ocean economy (Bennett, 2019; Satizábal et al., 2020). Coastal areas and marine resources are enclosed and privatised by state and private players, resulting in the displacement of peasant and fishing communities (Barbesgaard, 2018). Those with power often use the securitisation narrative to help secure nature, enclose it, and profit from it while disadvantaging the poor and less powerful (Kalina et al., 2019). Small-scale fisheries, for instance, are entangled in rationales of maritime border security (Song, 2021) as states safeguard their maritime space for blue economy developments (Childs & Hicks, 2019). Moreover, as exemplified in the port of Durban in South Africa, growth-driven objectives push states to employ securitisation narratives to marginalised subsistence fishers (Kalina et al., 2019; Maharaj, 2017).

The notion of accumulation by dispossession (Harvey, 2003) emanates from Marx’s concept of primitive accumulation. Marx’s notion of primitive accumulation described a historical phase of capitalist development, emphasising the origin of capitalist social relations and labour exploitation, particularly in the Global North (Roberts, 2020). Primitive accumulation has been expressed in several different conceptions, with accumulation by dispossession as one of the many variations of primitive accumulation (Hall, 2012). However, critics have observed that the varied conceptions of primitive accumulation impede its effectiveness. For instance, they argue that non-capitalist social forms are under-theorised (McCormack & Barclay, 2013), and there are disagreements about the boundaries of its characteristics, consequences, and intentions (Hall, 2013). Despite its shortcomings, the concept of accumulation by dispossession in this study provides a broad understanding of how market and growth-oriented relations produce exclusion in the process, particularly for the less powerful (Harvey, 2003; Prudham, 2007). It describes the processes and patterns of marginalisation and exclusion enabled by global capitalist development (Hall, 2013; Harvey, 2003; Roberts, 2020). It emphasises how capital accumulation prioritises the ‘rights of private property and profit’ over other rights, effectively dispossessing those who lack the means to accumulate (Harvey, 2008, p. 23).

The mechanisms of accumulation can be economic, as in capitalist social relations, or non-economic, as in the notion of accumulation by dispossession, which includes force and violence (Massé & Lunstrun, 2016). Ghana’s port development and industrialisation have taken a neoliberal route, with a quasi-state authority enclosing coastal areas, undertaking evictions, and enforcing securitisation. Both accumulation by dispossession or by securitisation can be situated within the primitive accumulation literature; however, the former stresses the motives of those accumulating, while the latter highlights the tools/mechanisms that enable the accumulation. As a result, this study employs these political ecology theoretical insights as a critical lens to understand how coastal transitions shape coastal fishing livelihoods in Ghana.

## Methods and materials

### Study area

With a population of about 30 million and a GDP of USD 67 billion, Ghana is classified as a lower-middle-income country (Ghana Statistical Service, 2021; World Bank, 2021). It has a total land area of 227,540 km^2^ and a coastline of 550 km (Bank of Ghana, 2008). Ghana’s continental shelf has approximately 225,000 km^2^ of maritime space, including a 200 nautical mile exclusive economic zone (Nunoo et al., 2014). Fish is essential to the Ghanaian diet and economy, with around 10% of Ghana’s population employed in the fishing sector (Sarpong et al., 2005; MoFAD, 2015).

**Fig. 1:**
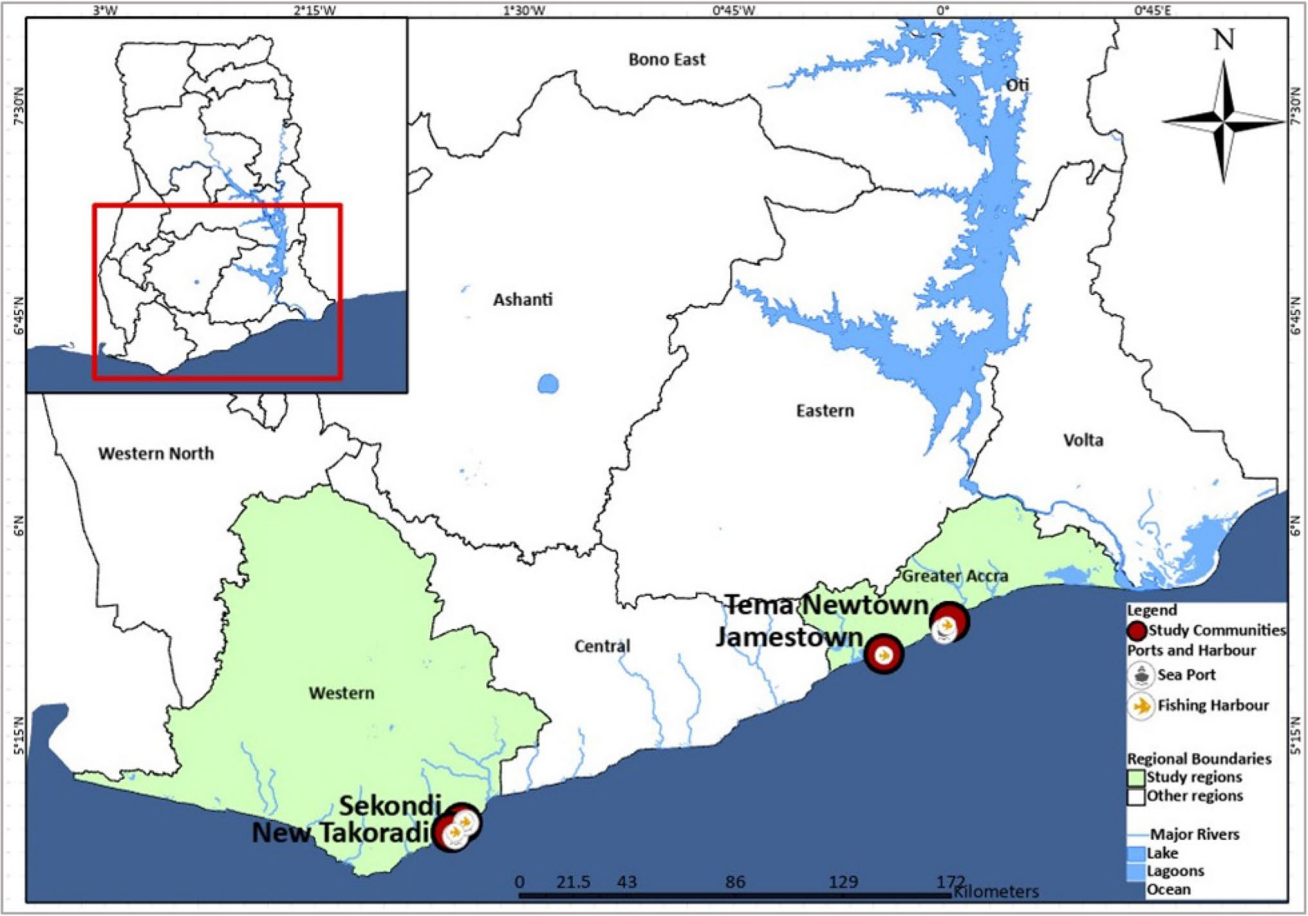
Map showing the location of study communities (Ayilu, 2023)

This research is conducted in the two Ghanaian coastal regions where the country’s commercial ports are located, the Western Region and the Greater Accra Region (Fig. 1). The four study communities — Sekondi and New Takoradi in the Western Region, and Jamestown and Tema Newtown in the Greater Accra Region — are urbanised areas. The Tema Newtown and New Takoradi communities are relocated settlements that gave way to the construction of the ports of Tema and Takoradi, respectively. Sekondi and New Takoradi are in the same local administrative district, while the other two are in separate administrative districts. Except for Jamestown, where there is ongoing harbour construction, all the other communities are adjacent to Ghana’s two main ports. Table 1 presents an overview of selected sociodemographic features of the communities studied. It is important to note that the high average catch in Jamestown may have been influenced by catches from other areas, such as Chorkor, Gbegbeyisee, and Osu, who mostly land their catches at Jamestown. Additionally, the catch data for Tema and Sekondi-Takoradi may not reflect the actual fishing effort and production in these areas, as some small-scale fishers may not record their catches or choose to land their catches in nearby communities.

**Table 1:** Selected characteristics of the study locations

Study communities	Tema Newtown	Jamestown	Sekondi	New Takoradi
Name of port/harbour in the community	Port of Tema	Jamestown Fishing Harbour Complex (Ongoing)	Sekondi Fishing Harbour	Port of Takoradi
District area	Tema Metropolitan	Accra Metropolitan	Sekondi-Takoradi Metropolitan	
Population of district	292,773	284,124	445,205	
Land area (km^2^)	396	200	664	
Number of local canoes	574	470	664	
Number of local fishers	5340	2981	4542	
Five-year average catch (MT)	4000	52,902	5000	
Main fish species	Anchovy (*Engraulis encrasicolus*), Sardinellas (*Sardinella* spp.), Bumper (*Chloroscombrus chrysurus*), Frigate Mackerel (*Caranx hippos*), and Chub Mackerel (*Scomber japonicus*)	Round Sardinella (*Sardinella aurita*), Bumper (*Chloroscombrus chrysurus*), Frigate Mackerel (*Caranx hippos*), and Flat Sardinella (*Sardinella* spp.)	Sardinella (*Sardinella* spp.), frigate mackerel, (*Auxis thazard*), and long-finned Herring (*Ilisha africana*)	

Source: Fisheries Scientific Survey Division (Dovlo et al., 2016); Ghana Statistical Service (2021)

The four communities were selected for the study because of their proximity to Ghana’s main ports. According to the Marine Canoe Frame Survey conducted by Ghana Fisheries Scientific Survey Division, the communities are active fishing destinations (Dovlo et al., 2016). The settlements are among the busiest of Ghana’s 186 coastal fishing communities, with fishing and related activities such as canoe manufacturing, net repair and drying, fish processing, and formal and informal fish marketing as the primary economic activities. In Sekondi and New Takoradi, for example, small-scale fishing accounts for nearly 85% of fishers’ monthly income (Owusu & Adjei, 2021). In the four communities, small-scale fishing actors faced similar disruptions in fishing livelihoods due to securitisation and dispossession related to port development, expansion, and operational activities.

### Data collection

The study used qualitative research methods involving focus group discussions and one-on-one interviews with local fishing actors and a port manager to address the main research question — how has access to and exclusion from coastal and maritime space influenced fishers’ livelihoods in Ghana port communities? The interview with the port manager was limited to one participant due to the difficulty of conducting research interviews with prominent elites — ‘studying up’ (Nader, 1969). The port manager is a government official from the Ghana Port and Harbour Authority, the institution responsible for managing the ports and harbours in Ghana. I also relied on information from the GPHA’s institutional report to supplement the field interview. The small-scale fisheries participants were chosen using a purposive strategy, which took into account their vast knowledge of community-based fisheries, marine livelihoods, and local fishing situations. Specifically, in Ghana’s coastal communities, the chief fisher (*apofohene*) and the chief fish trader/processor (*konkohemaa*) constitute the primary fishing decision-makers (Ameyaw et al., 2021; Bennett & Bannerman, 2002). These community leaders arbitrate disagreements among fishing actors and enforce local laws and customary management practices in their communities (Kassah & Asare, 2022). One-on-one, in-depth interviews were conducted with one chief fisherman and one chief fish processor from each community, making four chief fishermen and four chief fish processors from the four communities. In addition, five selected local fishers (men) and five local fish processors/traders (women) who serve on the local fishing committees were recruited to participate in two separate focus group discussions in each of the four fishing communities, resulting in a total of 12 people from each of the four fishing communities participating in the data collection.

The COVID-19 pandemic constrained the number of participants recruited for interviews because data collection in Ghana occurred during the second wave of the pandemic. Despite this limitation, the variety of small-scale fishing actors in each community represents the key decision-making actors who are well-informed about the community fishing concerns and developments. Overall, the study comprised 24 men and 24 women, reflecting the gendered labour division in Ghana’s small-scale fisheries value chain (Britwum, 2009). All the participants were recruited through contact during a community visit, followed by face-to-face interviews performed by a research assistant following an interview guide I developed. I took part in the field interviews remotely using Zoom technology and direct phone calls due to COVID-19 international travel restrictions at that period. We identified influential fishing community members as gatekeepers to overcome potential access barriers to conducting interviews with research participants. All research participants voluntarily consented to be interviewed, including being audiotaped. The interviews lasted between 30 and 90 min and were conducted in Ga and Fante, the indigenous languages of Greater Accra and the Western area. The interviews revealed a nexus between fishing communities’ exclusion from and access to coastal and maritime environments and the repercussions on their fishing-based livelihoods.

### Data analysis

The research assistant translated the Fante interviews verbatim into English, and the Ga interviews were translated into English through an interpreter. NVIVO-QSR International, a qualitative data analysis programme, was used to analyse the codes from the transcribed interviews. Familiarisation with the data allowed for the identification of themes. The transcripts were read multiple times before coding, and then the codes were re-evaluated and validated again by reading codes against the original transcripts. The multiple readings of transcripts ensured that the data was thoroughly understood before coding, and the validation of codes by re-reading them against the original transcripts provided an additional check to ensure the accuracy and reliability of the codes. This process helped ensure that the codes generated accurately reflected the content of the transcripts and that the results accurately reflected the data collected. Using a case study methodology (Yin, 2013), the four communities are grouped into two case studies to form the findings section: (i) the Jamestown fishing harbour complex and (ii) the ports of Tema and Sekondi-Takoradi. The context-specific advantage of the case study approach aligns with the political ecology theoretical themes used for the study. The study also utilises peer-reviewed and grey literature and media reports to support the findings and discussions.

## Research findings

### Case study 1: the Jamestown fishing harbour complex

**Fig. 2:**
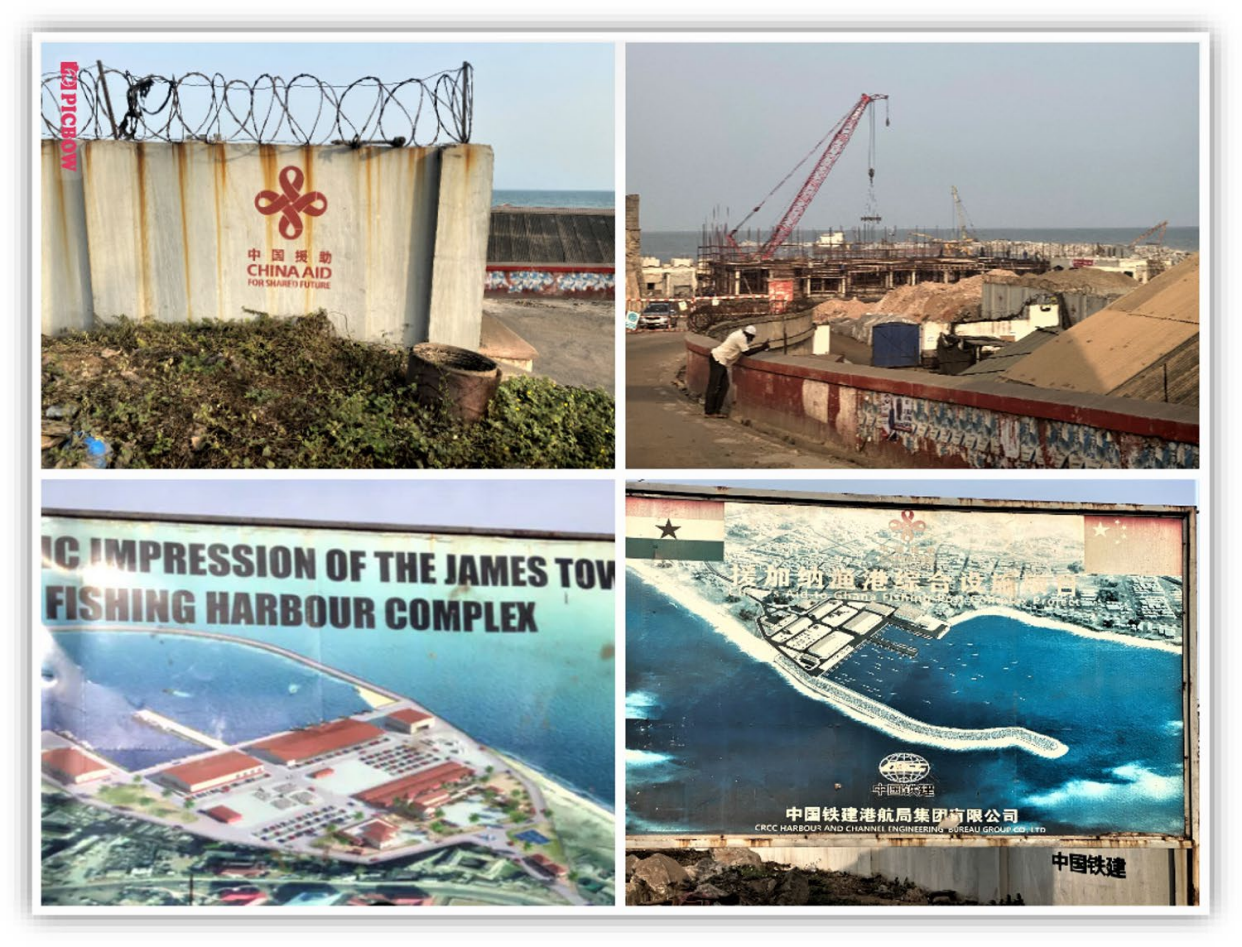
Images showing construction work in progress at the proposed Jamestown Fishing Harbour Complex and artistic impressions of billboards around the construction site. Source: Ayilu (2023)

Following the gold and cocoa boom of 1879, the British colonial administration constructed the Jamestown port to provide docking space for smaller surfboats that ferried goods in and out to the ships anchored in deeper water (Malkoc, 2020). In the 1960s, the Jamestown port was decommissioned with the development of a modern port — the Port of Tema. However, it remains a relevant site for the small-scale fishing livelihoods of the approximately 2000 inhabitants in the Ga-mashie neighbourhood. This community has used the harbour area for small-scale fishing activities, including canoe docking, construction, fishing equipment repair, and smoking and trading of fish (Malkoc, 2020). In 2018, the government of Ghana initiated state-sponsored demolitions in the area to develop a fishing harbour complex, sparking broad contestation by the fishing communities (Citi Newsroom, 2021a; VOA, 2020). Supported by USD 50 million from China Aid, the Jamestown Fishing Harbour Complex (Fig. 2) involved dredging a harbour basin of 100,000 m^3^ and constructing hydraulic structures and other facilities covering an area of 13,000 m^3^ (Citi Newsroom, 2021a; Graphic.com.gh, 2020). The harbour also includes fish landing sites, cold storage facilities, market areas, and other social amenities. Those backing the new harbour project emphasised that it would modernise Ghana’s fisheries, including small-scale fishing, and maximise the country’s fishing potential, by creating about 1000 job opportunities in the local community (Dredging Today, 2018). In this case study, the interviewed local fishing actors in Jamestown described significant disruptions to local fishing, fish smoking, and small-scale fish trading as a result of the new port project.

#### Displacement of small-scale fishing actors

Jamestown is an important fishing community in Ghana, with fishing activity dating back to the sixteenth century. The local population comprises the Ga people and migrant workers from Ghana and other West African countries (Wrigley-Asante & Mensah, 2017). Fishers, fish traders, and processors in Jamestown live and work together in a small neighbourhood called Ga-Mashie. The Ga-mashie fishing neighbourhood has an intimate relationship with the sea that is dictated by its connection to fishing. On the beach are businesses, dwellings, schools, worship centres, and drinking bars; the fishermen consider the neighbourhood to be their home.

From 2015 to 2018, the Accra Metropolitan Assembly (AMA) repeatedly brought bulldozers and military personnel to demolish the fishing community’s buildings to get the fishing population, who mostly lack tenure rights, to evacuate the area. Since the first round of demolitions in 2015, the cycle of residents returning to the port following each demolition occurred at least three times (Malkoc, 2020). In 2018, the AMA successfully demolished the buildings and other properties of the fishing community, making way for what the government described as the ‘Jamestown Fishing Harbour Complex’. However, these state-sponsored demolitions have displaced local fishing actors and disrupted traditional coastal fishing livelihoods. The local fishing actors have raised concerns about the demolition of both temporary and permanent structures, including schools and churches. The fishers, traders, and processors could not relocate their businesses and property before the AMA demolished the neighbourhood. According to the participants, the AMA forcibly evicted them by deploying state security forces before demolishing their structures without notification, a claim that has been disputed by the AMA (The Ghana Report, 2020). The local authority insisted that only ‘illegal structures’ were demolished and that they gave adequate warning to the small-scale fisheries actors, but they refused to cooperate (Citi Newsroom, 2021a). In the media, some of the fishers reportedly confirmed that the AMA met with them before the demolition (The Ghana Report, 2020). Small-scale fishing households in Ghana mostly lack tenure rights to seafront landing areas, as the lands belong to either individuals or the local community chief (FAO, 2021). In addition, Ghanaian law does not grant illegal squatters or settlers the right to compensation unless they can demonstrate a legal claim to the property (West Africa Regional Fisheries Program, 2011). One of the local fishers explained their frustration:

As poor fishers, we have been deprived of our land and means of subsistence. The Jamestown neighbourhood was all we had, and we did everything there, but the government and the politicians have driven us out of our own community as if we were criminals because of the harbour project. (Interview #2, a 50-year-old fisherman)

The AMA relocated the fishing actors in the Jamestown community to a new beach area approximately 2 km east of the original location. The new location is next to a site proposed by the Ghanaian government for a business and recreational project — Marine Drive Project (Citi Newsroom, 2023), which small-scale fishers and traders claim is temporary. They contend that the area is unsuitable, lacks appropriate shelter, and is too rough for canoe landings, as fishermen routinely report gear damage and potential canoe capsizes. In addition, the seafront lacks a berthing point, cannot accommodate the community’s large number of canoes, and is unsuitable for repairing fishing nets and building canoes. During the data collection, some of the issues raised by the fishers were visible at the location. Fishers fear they may lose the beach area to the recreational project and may not be admitted back into the harbour when it is completed. One of them explained:

The government might ask us to leave our community beach forever, but we will keep fighting. We are aware that more Chinese trawlers are being introduced into our waters every day by these same politicians and that they may require docking space. I am afraid that the new fishing port complex project is for trawlers and other big fishing companies. (Interview #1, a 56-year-old fisherman)

According to the fishers, most of the displaced fishers’ have migrated to other communities with few crew members, leaving most of the crew in the original community without a source of livelihood. Small-scale fishing in Ghana is a labour-intensive endeavour, with crucial pre- and post-harvest activities requiring a substantial labour force. A single canoe may employ up to eight or more crew members. Over the years, the fishing operations and arrangements have provided the community with the necessary employment that has now been interrupted by the new harbour project.

Furthermore, the local fish processors and traders contend that their smoking ovens and sheds were demolished and subsequently relocated. They explain that the current location is temporary because of the nearby recreational development project. As a result, fish processors are reluctant to invest in permanent ovens and sheds due to uncertainty regarding their tenure and future. They argue that the new location’s landscape is also unsuitable for the smoking, drying, and selling of catch, which they argue impacts the ability to process catch. In order to preserve the freshness of the fish, processors and traders incur additional costs for freezing fresh catches and procuring improvised containers. Additionally, traders have also stressed the problem of customer attrition, as they have observed a significant drop in clients due to their inability to access new locations. A sizable proportion of urban customers (backpackers, expatriates, and the hospitality business) who relied on them for fresh seafood are lost to cold store operators in the city. A processor summarised:

[The local authorities] refused to make any arrangement for us in this new space… we have lost our men who give us fish, our customers who buy our fish and we have also lost our processing facilities. (Interview #8, a 47-year-old processor)

According to the processors who participated in this study, fishers have migrated elsewhere to form new alliances in other fishing communities, depriving them of the necessary catch volume to process, market, and finance more fishing expeditions. The processors and traders argued that they cannot finance more fishers to increase the volume of catch landings, as the once-profitable fish trading business is now struggling due to a lack of sufficient catch. They emotionally recounted their social and physical disconnects from fishers, with whom they had built trusted relationships to maintain reliable fish supplies. Both local fishers and traders unanimously contended that Jamestown had lost its prominence as a tourist destination due to the disruption of the community’s thriving fishing culture. Jamestown is a famous beach destination in Ghana’s capital, where tourists observe indigenous fishing culture, distinctive scripted canoes, and vibrant traditional fish smoking practices using an oven called ‘Chorkor Oven’.

### Case study 2: the ports of Sekondi-Takoradi and Tema

Sekondi-Takoradi lies in the West Region, 200 km from Accra, the capital of Ghana. The communities began as two fishing settlements located a few kilometres apart and eventually developed into important trading centres due to their proximity to each other (Yankson et al., 2017). The Port of Sekondi-Takoradi, including the fishing harbour, was constructed in 1928. Since Ghana’s independence in 1957, it has been expanded with additional berthing construction and refurbishment, particularly during the Economic Recovery Programme. As of 2020, the vessel carrying capacity in the Port has grown, handling 28% of national seaborne traffic, 17% of national seaborne imports, and 64% of exports (GPHA, 2022). Since Ghana discovered oil in commercial quantities in 2007, the Port of Sekondi-Takoradi has become the hub for offshore supply vessels in the Jubilee Oil Fields off the Western Region at Cape Three Points. The Port of Sekondi-Takoradi breakwater current extends 2.7-km long, with an 800-m bulk jetty under construction, a 590-m quay wall, and a 16-m-deep berth pocket (GPHA, 2022). The Port has focused on infrastructure expansion and facility upgrades to maintain a competitive edge in the West African subregion’s oil and gas sector. Alongside the Takoradi port lies the Sekondi fishing harbour, which was recently renovated with financing from the Japan International Cooperation Agency (JICA) (Gyan et al., 2020). In addition to its two piers, the Sekondi fishing harbour is equipped with an ice-making plant, an ice storage facility, and a fish-handling and marketing area (Gyan et al., 2020).

The Port of Tema is the largest deep-water seaport in Ghana, located about 29 km east of the capital, Accra. Commissioned in 1962, the Port of Tema consisted of two breakwaters that enclosed an area of 500 acres, including 12 berths, cocoa sheds, a dry dock, a slipway, a workshop, and offices. In 1964, it expanded with the development of a new shipyard complex and the acquisition of an additional 64 m^2^ of land north of the Port of Tema for workers’ residential housing. With the robust economic growth and rapid maritime trade in Ghana and the subregion, particularly in the northern Sahelian countries, the port managers developed new container terminals. To accommodate increased container traffic, the Port has expanded westward since 2010. The port operation has also undergone significant restructuring, including increased private-sector participation, with some considerable port services now privatised. An important part of the Port of Tema is the Tema Fishing Harbour. The harbour is designed to accommodate a wide range of fishing vessels, from small boats used by local fishers to large trawlers operated by industrial fishing companies.

This section discusses how the rising privatisation of certain portions of the Port, along with port expansion and continued development, has resulted in substantial securitisation at and around the Port, and negatively impacted the fishing community, including socio-cultural dimensions. This study considers the two existing seaports in Ghana (Sekondi-Takoradi and Tema) as a single case because they have experienced similar developments, and the impacts and disruptions to the coastal fishing communities are comparable.

#### Securitisation and port expansion

**Fig. 3:**
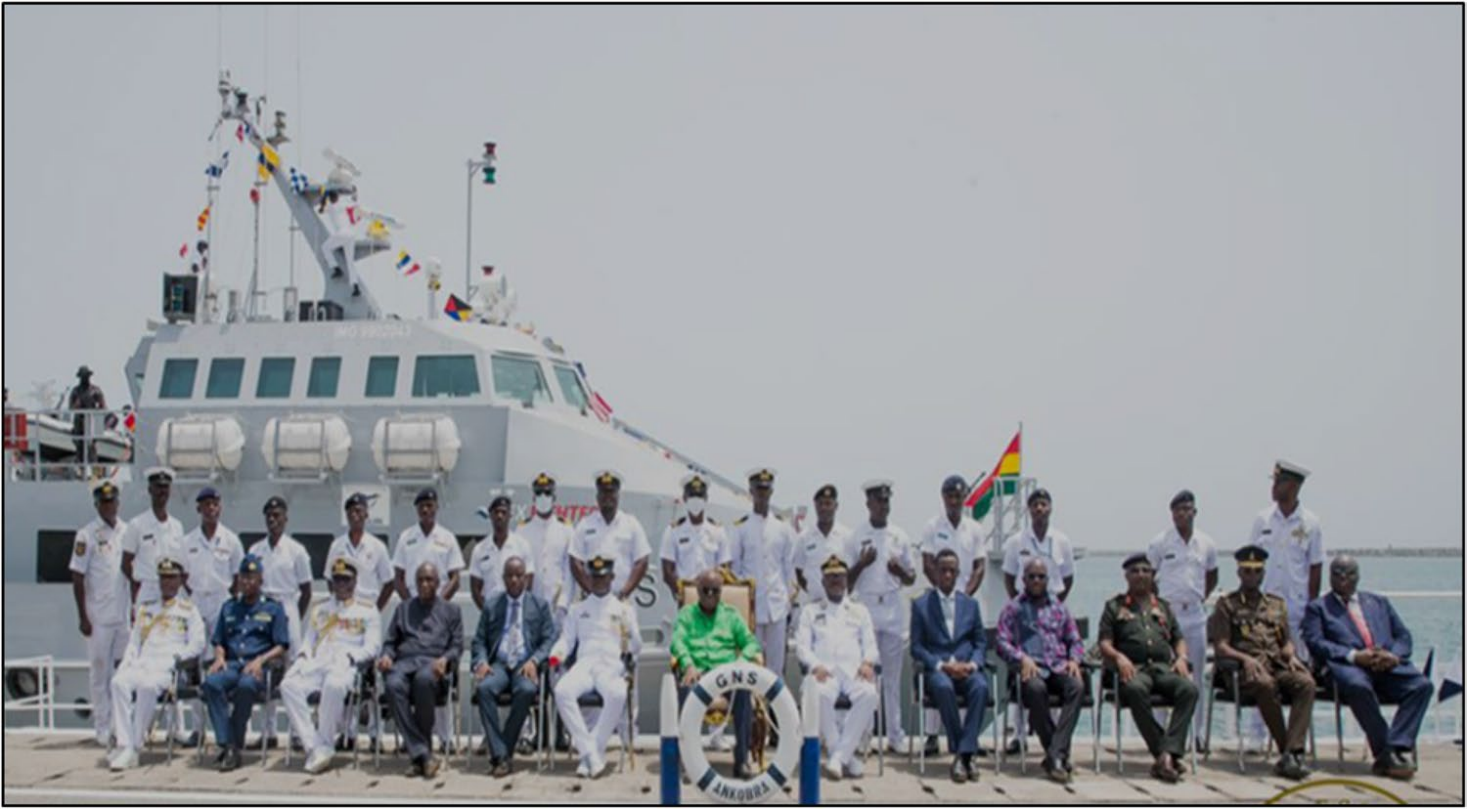
The President of Ghana, Nana Akuffo Addo (middle of the first row), 2022 commissioned four operational ships for the combating of illegal maritime activities. Source: Myjoyonline.com, 2022

Successive Ghanaian governments have invested considerably in maritime security as part of the country’s blue economy modernisation and growth-driven efforts amidst the increasing cases of pirate attacks along the coastline (Business and Financial Times, 2021). In 2011, the previous National Democratic Congress government acquired four Chinese-made patrol ships to bolster Ghana’s maritime security, citing the need to protect ‘the country’s territorial integrity and provide safe sea passage for legitimate traffic while combating illegal activities’ (Stop Illegal Fishing, 2011). In addition, the current ruling New Patriotic Party government commissioned four Israeli-built special security vessels in 2022 (Fig. 3) to combat illegal maritime activities on Ghana’s coastline (Citi Newsroom, 2021a; Myjoyonline.com, 2022). According to the President of Ghana, Nana Akuffo Addo, the intervention will ‘protect [the] maritime domain to boost the blue economy sectors, which include shipping, fishing and offshore oil and gas production’ (Citi Newsroom, 2021b).

The GPHA, a quasi-state authority, has implemented safety and security measures to secure the operational environment and installations in the ports of Tema and Takoradi. According to GPHA, these measures aim to guarantee the safety of investments within the ports and to bring the ports into compliance with the International Ship and Port Facility Security Code (ISPS) (International Maritime Organisation [IMO], 2003). The GPHA emphasises that seaport expansions and efficiency would significantly propel the country’s economic growth potential, benefiting the local fishing communities through increased employment (GPHA, 2022). However, small-scale fishing participants interviewed in the port communities have raised concerns about their precarious livelihoods due to continuous securitisation and expansion development. In pursuing such securitisation measures and growth-oriented expansions of the ports, the coastal fishing communities are progressively excluded, marginalised, and displaced. Port officials prohibit fishing in proximity to some specific zones, including the expansion area and docking zones of cargo and industrial fishing vessels, resulting in tension between the port authorities and local fishers. According to the participants in Tema New Town, Sekondi, and New Takoradi, port authorities have frequently cited security concerns in confiscating their fishing equipment. They claim that fishing areas have been reduced because a large portion of the ocean’s surface has been securitised, limiting their access to near-shore fishing grounds due to port expansion and operations.

The fishers oppose the security arguments of the port officials and allege that they had an understanding with them to fish in those areas periodically. They claimed that vessel food residue attracts fish to those locations, and the prohibitions deprive the local fishers of the necessary catch. In Tema and Sekondi-Takoradi, fishers claim that there are now stringent restrictions and arrests of local canoe operators in the Port. The port official who participated in the research argued that the decision to restrict the fishers to specific zones of the Port is because Ghana risks international sanctions if local fishing activities affect the port operations. The port manager explains:

Fishers frequently use local canoes for illegal activities, and there have been instances in the past involving illicit drugs violating international port regulations. You cannot take a foreigner (tourists) from the canoe basin or load items to and from the vessel because everything needs to be inspected. (Interview #10, a 49-year-old Port Manager)

However, local fishers have opposed the port manager’s argument, stating that the decline in fish catches has driven some of them to transport tourists and provide chandler services in order to make a livelihood.

Since the construction of the Sekondi and Tema Fishing Harbours, the port authorities have zoned them. The Tema Fishing Harbour has four main zones: an outer and inner fishing harbour for industrial vessels, a canoe bay for traditional fishing activities, and a commercial area for fish trading. Similarly, the Sekondi Fishing Harbour comprise two piers, local fish handling bay, and a commercial shed, including an ice-making and storage plant. In the commercial areas of both fishing harbours, women sell both locally harvested fresh fish and frozen fish from nearby cold stores. The GPHA controlled the fishing harbours, including the canoe bay area, which the port authority considers a form of corporate social responsibility to the local fishing communities. The daily activities in the canoe bay are managed by the chief fishermen, who serve as leaders of the fishers. There is limited space to expand the canoe bay due to the presence of port installations designated as security zones. The increased fishing population in the communities over the years has led to congestion at both the Tema and Sekondi-Takoradi fishing harbours. As a result, fishers are squeezed into the canoe bay, which is affecting how local fishing is organised. Local fishers in Tema, for instance, reported at peak season, particularly during the COVID-19 pandemic, when social distancing procedures were enforced, they queued up to unload their catch. Likewise, at the Sekondi Fishing Harbour, the limited docking space available in the canoe bay has become a source of conflict for small-scale fishing actors. The high volume of industrial fishing vessels also leads to congestion, which causes damage to canoes and fishing gear. A fisher explained:

This space in the Port [referring to the canoe basin in Tema] is now too small for us. The number of boats here is way too many, so we wait for long hours before you can get space to offload your fish, which reduces the quality of the catch and our profit margin. (Interview #18, a 42-year-old fisherman)

Port securitisation does not disrupt industrial fishing activities in the same way as the small fishers because they are better equipped and can conduct deep-sea fishing undisrupted. In the Sekondi port, for instance, small-scale fishers contend that the breakwater and piers at the canoe basin are unsuitable for their small canoes, thus giving the industrial and semi-industrial vessels a docking advantage over them. The local fishers in Tema and Sekondi have accused port authorities of denying them tenure rights to the designated canoe basins in the port area. Local fishers demand that they govern the canoe bay themselves, as is the case with other fishing communities, which they believe would stop the constant threats by the port authorities and potential future eviction. A chief fisher explained:

Our voices are neglected, but we are affected by every action in this Port. Since the port authorities are in charge of this canoe bay, when we meet with them, we only receive orders and instructions regarding where we may and cannot fish. (Interview # 16, a 54-year-old fisherman)

In addition, the significant increase in cargo and industrial vessel movement has reduced fishing space at and around the Port. The fishers also complained of considerable vessel traffic occasionally overflowing the fishing zones and the local fish-landing bay. Some local fishers, who cannot afford the high fuel cost and crew to travel far for fishing trips, choose to forego fishing or relocate from the community. A fisher said:

As fishers in this community, we invest additional time and fuel travelling to fishing locations outside of the port area in order to avoid accidents with big vessels. Even at sea, we feel the congestion; we hardly get the space to cast our nets, so we have to keep going further and farther away from land. (Interview #25, a 39-year-old processor)

Furthermore, the government has compulsorily acquired large portions of the communities’ land to develop the Tema and Sekondi-Takoradi ports. In contrast, the fishing communities are squeezed into densely populated neighbourhoods. Local fishers in Tema New Town, Sekondi, and New Takoradi argue that rapid urbanisation due to the port development is uneven as they are left behind. Initial communities’ settlements occurred decades ago when residents had to be relocated for the ports to be built. While the communities remained formally part of the busiest urbanised port cities due to proximity, fishers are practically disconnected from the fortune of urbanisation, including essential community services such as access to clean water and proper drainage. The fishers explained that becoming part of port cities exposes them to urban economic pressures, including rising food costs and rent, making it difficult to meet these basic needs with their fishing incomes. For example, small-scale actors attribute Tema Newtown’s degeneration into a slum to the high cost of rent, blaming it on the government’s decision to locate the Port on their ancestral land. Similar claims have been made by the International New Town Institute (2022), attributing the transformation of Tema Newtown into a slum to the port development. The effects of port development on coastal communities are a global issue. Such developments often result in a loss of traditional livelihoods, displacement, and a decline in the quality of life for fishing communities (see Okafor-Yarwood et al., 2020).

Furthermore, biophysical interactions, such as extensive dredging for port expansion, have influenced the socio-cultural dimensions of the fishing communities. Specifically, the entire beach area in New Takoradi has been converted into a sea defence wall, depriving the residents of beach space for recreation. Also, the local fishing actors argue that the presence of the ports eroded their traditional and cultural values. They contend that industrial businesses, shipping vessels, and large fishing trawlers in the ports disregarded the long-standing tradition of refraining from going to sea during specific periods of the year, especially during coastal fishing festivals. In particular, fishers in Tema New Town also lament the damage to a spiritually significant stone on the coast due to port expansion.

## Discussion and conclusion

This study is based on empirical research conducted in Ghana, drawing on case studies organised according to two forms of marginalisation experiences: dispossession and securitisation. The study unpacks how the notion of accumulation by securitisation and dispossession occurs in practice through the experiences of small-scale fishing actors in Ghana’s port communities. The shift to the blue economy has opened up the ocean and coastlines for social-material transformations, including port development, raising equity issues in the distribution of maritime resources (Barbesgaard, 2018; Bennett et al., 2019) that require further theoretical enquiry. The study has demonstrated that the growth-oriented imperatives of new ports or large port expansion and development in Ghana have lost sight of the potential impacts of exclusion and marginalisation on local coastal livelihoods. These forms of accumulation leading to dispossession, especially for fishing livelihoods, have been extensively documented in maritime frontiers (Bavinck et al., 2017; Kalina et al., 2019; Maharaj, 2017), sometimes described as coastal (Bavinck et al., 2017) or ocean (Bennett et al., 2019) ‘grabbing’. Although a less explicit depiction of neoliberal expansion, this study contends that ocean/coastal grabbing can be situated within the primitive accumulation literature as another crucial mechanism of neoliberal dispossession.

The Government of Ghana, through the GPHA, has initiated large-scale infrastructure development to allow the ports to receive larger vessels, handle more cargo, increase storage capacity, reduce the cost of trade, and thereby increase Ghana’s regional trade capacity (African Development Bank [AfDB], 2017). Ghana’s ambition to be the maritime gateway in the West African sub-region and to boost its mineral exports has resulted in the expansion of the Port of Tema to become the preferred Port for the neighbouring Sahel countries, and the Port of Takoradi to become the West African hub for the emerging oil and gas industry services (GPHA, 2022). However, in the process, the securitisation of port areas has enclosed access to coastal and ocean spaces, and fishing resources depended on by nearby fishing communities, substantially affecting their fishing livelihoods. Additionally, in the Jamestown area, a new harbour project that aims to modernise and harness the country’s fishing potential has exacerbated the displacement of small-scale fishing livelihoods.

Small-scale fishing livelihoods in the rapidly transformed urbanised port communities of Tema Newtown, New Takoradi, and Sekondi are entangled in complex social, political, and material processes, contrary to the common assumption in the conservation science literature that small-scale fishing actors are concerned with declining fish stocks and the necessary technology needed to secure catch (Kadfak & Oskarsson, 2020). Emerging transformations associated with the urbanisation of the community, economic growth objectives through port developments and expansions, and port governance measures have resulted in unstable livelihoods for the adjacent fishing communities. Local fishing communities have been excluded from their traditional fishing grounds and locations, with Ghanaian port authorities claiming that they are implementing security measures in the port area in line with international standards (Kalina et al., 2019; Maharaj, 2017). Similar security risk narratives have been advanced against small-scale fishers in oil and gas production communities in the Western Region of Ghana by state officials and private companies (see Adjei & Overå 2019). The GPHA exercises much discursive power, portraying small-scale fishers as ignorant, illiterate, traditional, and irresponsible to justify securitisation actions. For instance, the GPHA continues to manage the canoe bays in Ghana’s fishing harbours instead of allowing the small-scale fishers to make their own operational decisions. In addition, fishers assert that they are minority stakeholders in the port community whose opinions are easily disregarded because port officials consider them poor fishers. The perception of branding fishers as backward and small-scale fishing, in particular as synonymous with poverty, has been documented in the literature (see Adjei & Overå 2019; Béné, 2003). Local fishers interviewed argue that the strict restrictions imposed by the port officials are because the government prioritised commercial investments and port activity over them. This top-down view is reproduced even when local fishers are included in the decision-making processes in the ports, such as the management of the local canoe basin where they operate. This discourse shapes the imbalanced power relations between port authorities and small-scale fishers when the fishers negotiate access to fishing livelihoods in the communities around Ghana’s two main ports (Maharaj, 2017).

Likewise, in the Jamestown community, the ongoing harbour project has become a site for contestation between small-scale fishing actors and local government authorities. From the 1960s until 2018, when a new fishing harbour project started, the old Port and surrounding coastal zones have been public spaces for fishermen in the community. The ongoing contestations are based on how each interest group perceives the present status of such coastal land in the Jamestown community. As Lund (2013) emphasised, the complexities of tenure interpretation deployed by different groups to support land claims are asserted in multiple interpretations and conceptions. These conceptions and interpretations of tenure, which may include ancestral rights, customary land title, or the legalisation of land authority by the central government, all serve to consolidate some forms of access, ownership, and control over land while excluding others (Andrews, 2018; Lund, 2012; Peluso & Lund, 2011). As noted by others, depending on the present prevailing conditions and the future ambitions of particular groups of players in land disputes, aspects of the ‘past’ could be reconstructed in an attempt to reclaim land (Kansanga et al., 2019; Knudsen, 2012; Lund & Boone, 2013).

In Ghana, the colonial government acquired the Jamestown coastal land from the traditional landowners in the late 1900s and developed the old Port. However, after constructing the Port of Tema in the 1960s, the government decommissioned the Jamestown port, and the community reclaimed the coastal land for small-scale fishing activities. Despite no official land title transfers supporting the local fishing players’ reclamation of the coastal land in the community, they now proclaim their ancestral rights to the land. In contrast, the government, through the local authorities, has declared the fishers in the Jamestown harbour to be squatters who lack economic and political rights to the coastal land. Furthermore, land tenure and user rights claims have emerged as critical concerns for urbanising fishing communities as urban economic development projects attempt to claim regions historically reserved for traditional fishing (Fabinyi, 2020; Fabinyi et al., 2022; Kadfak & Oskarsson, 2020). In Ghana, for instance, Denchie et al. (2021) illustrate how the growth of the petroleum sector has transformed land use and access, resulting in user disputes between investors and local people. In Jamestown, the local fishing actors are sceptical about the construction of the new fishing harbour because they are concerned about losing the community’s coastal zone to other influential players, particularly industrial fishing trawlers.

Harvey (2003) argued that the ruling and political class consolidate power by dispossessing the less powerful of assets and livelihoods in order to accumulate wealth. In Southeast Asia, for example, studies have found that tourism and beachfront property development by foreigners and local elites obtain the most benefits, whereas marginal groups of fishers are vulnerable to displacement (Fabinyi et al., 2022; Knudsen, 2012). In Africa, a similar situation is observed by Benjaminsen and Bryceson (2012), where rent-seeking state officials, transnational conservation organisations, and tourism companies benefit from wildlife and marine conservation while excluding the local people. In the case of mega-investment projects like the James Town Fishing Harbour Complex, Chinese and Ghanaian elites engaged in large industrial fishing can potentially accumulate more profits, displacing the local fishers from their assets and livelihoods (Malkoc, 2020). The local fishers interviewed, for instance, pointed to the artist’s impression of the new fishing harbour complex, which does not show any local canoes. Instead, it depicts a reconstructed harbour with new shipping lanes, berths, seawalls, a breakwater, and facilities to support industrial fishing, such as a fish-processing centre and market (see Fig. 2). Others have described the new harbour complex project as nothing short of a ‘mechanised fish factory backed by China’, as the Chinese government is financing the project (Jackson, 2019). On the other hand, the government draws on the modernisation narrative to legitimise the deployment of exclusionary actions such as repeated force evictions and demolitions (Hall et al., 2011). However, as argued by Overå (2011), the modernisation of Ghanaian fisheries has supported the rise of industrial trawlers relative to local small-scale fishing, significantly disrupting the local coastal fishing value chains. In the context of the Jamestown community, the government narratives and the fishing actors’ traditional livelihood concerns present two competing interests, opposing ideas, and contested interpretations of facts.

To conclude, while the blue economy discourse underlines the need to balance competing interests in marine resources and spaces, its elusive character raises concerns about what blue growth means for diverse people and communities. A critical component of the blue economy is the growth-driven imperative that promotes the commodification of natural resources and the growing territorialisation and securitisation of the ocean and coastal spaces by competing interests, including port developments, industrial/economic zones, and tourism (Barbesgaard, 2018; Childs & Hicks, 2019; Fabinyi et al., 2022; Kadfak & Oskarsson, 2020). Small-scale fisheries livelihoods are increasingly entangled in these diverse coastal transitions and developments. Notably, urban coastal fishing livelihood opportunities are increasingly constrained by the broader economic and political dynamics and trends along the coast. As demonstrated in Ghana, port developments, expansions, and operations have facilitated ocean/blue grabbing and the exclusion of urban coastal fishing livelihoods. The expansion of ports is facilitated through actions ranging from eviction and demolition to access restrictions. These actions are underpinned by Ghana’s economic growth-oriented objectives to enhance international maritime trade, industrialise and become an oil and gas industrial centre in West Africa, and harness the country’s fishing potential.

This study builds on the growing political ecology scholarship that questions the growth-oriented expansion in the blue economy (Barbesgaard, 2018; Bavinck, 2017; Ertör & Hadjimichael, 2020; Kadfak & Oskarsson, 2020). Barbesgaard (2018) argued that neoliberal tendencies drive the blue economy revolution with the potential to threaten coastal populations. This study demonstrates how the diverse forms of marginalisation impact urban coastal fishing actors’ access to meaningful livelihoods in Ghana. This has potential impacts on food security because fish traders in coastal communities distribute fish products to large metropolitan markets in Ghana and other neighbouring nations (Ayilu & Niawung, 2022; Overå et al., 2022). In answering the question of how accumulation by dispossession and securitisation shapes urban coastal fishing livelihoods in Ghana, this study makes three contributions. First, by accentuating the voices of urban coastal fishing actors in Ghana, it demonstrates that securitisation is a crucial non-economic mechanism of accumulation that enables those who perpetrate the ocean/blue grabbing (Benjaminsen & Bryceson, 2012). In the blue economy, securitisation is connected to marine spatial planning that aims to reconcile the numerous competing interests in the ocean sectors (Josse et al., 2019). Yet, in practice, marine spatial planning is supported as a strategy to provide a stable maritime and coastal environment for blue economy industries (Flannery & Cinnéide, 2012). Second, the study augments other theoretical contributions by arguing that ocean/blue grabbing could be analytically positioned within the literature on primitive accumulation and accumulation by dispossession (Bavinck et al., 2017: Benjaminsen & Bryceson, 2012). Third, the study draws a deeper connection between urban small-scale fishing and the phenomenon of growth-oriented accumulation in the ocean economy frontier. Despite the growing political ecology literature addressing the marginalisation of small-scale fisheries, there is still a paucity of case-based empirical research highlighting the specific exclusions of urban coastal fishing communities and their consequences. This study contributes to bridging this empirical gap by illuminating how urban small-scale fishing actors are entangled within the sociopolitical and structural processes of coastal transition in their geopolitical and socio-cultural contexts.

In conclusion, ports are vital for economic growth, yet their development, expansion, and operations can negatively affect access to crucial fishing resources and livelihoods, as demonstrated in this study of coastal fishing in Ghana. As coastal small-scale fishing actors lose their only source of livelihood in the face of securitisation and displacement, there is potential for resistance, which may lead to different forms of maritime conflict (Pomeroy et al., 2016; Spijkers et al., 2019). Therefore, the study recommends that Ghana’s government address these exclusions to ensure that small-scale fishing actors are not marginalised by its adoption of the blue economy.

## Data Availability

Due to the research participants’ confidentiality and privacy concerns, the datasets collected for this research may not be made publicly available except in limited circumstances.
